# Isolation of *Acanthamoeba* Spp. from Drinking Waters in Several Hospitals of Iran

**Published:** 2010-06

**Authors:** HR Bagheri, R Shafiei, F Shafiei, SA Sajjadi

**Affiliations:** 1 Dept. of Microbiology, Center of Research Cellular and Molecular Medicine, Baqiyatallah University of Medical Sciences, Tehran, Iran; 2 Dept. of Medical Parasitology and Mycology, Shiraz University of Medical Sciences, Iran; 3 Health Center of Khorasan Razavi Province, Iran; 4 Dept. of Environmental Health, Gonabad University of Medical Sciences, Iran

**Keywords:** *Acanthamoeba*, Water, Hospital, Iran, Protozoa

## Abstract

**Background:**

*Acanthamoeba* is an opportunistic amphizoic protozoan found in different water sources including swimming pool as well as in sewage. The aim of this study was to investigate the prevalence of *Acanthamoeba* in tap-water samples in Iran.

**Method:**

In this descriptive cross-sectional study, 94 samples of cold and warm tap-water were collected from different wards of hospitals in 13 cities of Iran in 2007–2008. Free residual chlorine, pH, and temperature of samples were measured. After filtration through multipore nylon membrane, samples were cultured on non-nutrient agar. Then we investigated existence of *Acanthamoeba* by reverse contrast phase microscope.

**Results:**

*Acanthamoeba* was found in 45 samples (48%). Thirty-four and 11 positive samples were collected from cold and warm tap water, respectively. The samples belonged to the category of 20–30°C temperature with 0–2 ppm free residual chlorine and pH 6–7.4 showed the most coincidence to the positive cases. The greatest proportion of positive samples was obtained from Mashhad hospitals, while all samples collected from Arak and Semnan hospitals were negative.

**Conclusion:**

considering the results of this study and the pathogenic role of this protozoan on patients with immunodeficiency, as well as capability of this microorganism in carrying other pathogens such as *Legionella*, further studies are needed. What is more important, potable water in hospitals should follow the procedure of treatment and sanitation, in order to prevent the relevant nosocomial infections.

## Introduction

A *canthamoeba* is an opportunist amphizoic protozoan, which is found in the environmental sources. Researches showed that *Acanthamoeba* can be found in quite different media including sea water, treated water, swimming pool, aquarium, bottled water, soil, air dust, sewage water, contact lenses washing solution, food stuff, air conditioners, digest organs and dialysis machines (1). This protozoan has two stages in its life cycle, active trophozoite, and resistant cyst. The double-layered coat of cyst enables it to survive in the presence of disinfectants, such as chlorine compounds and antibiotics. It also well tolerates rang of temperature as wide as −2°C to +45°C. A variety of microorganism, such as *Legionella* sp. and *Burkholderia picketti,* which nest in the form of endosymbiont in this amoeba as amoeba-associated bacteria, can also survive after chlorination and applying other disinfectants (2–5).

Pathogenicity of *Acanthamoeba* was discovered by Culbertson and his colleagues in 1985 (6). This protozoan can enter the human body in the form of cyst or trophozoite via polluted water, soil or air (2). Several studies have reported that *Acanthamoeba* can attack to central nerve system (CNS) leading to granulomatous encephalitis. Acanthamoeba also target other organs such as eye, which end up with amoebic keratitis, as well as skin lesions in the patients with immunodeficiency, and in healthy individuals (7). It was also found in the upper respiratory tract, in apparently healthy persons as natural flora. Most of keratitis cases have shown a history of swimming in pool or sea. In addition, some cases have been reported amongst people applying unsterile solutions such as drinking water for cleaning their contact lenses (1, 8). This type of keratitis led to uveit wounds, severe eye pains (Keratonoritis), photophobia and blindness (9).

Polluted water with *Acanthamoeba,* which is improperly used for washing and disinfecting contact lenses, is generally due to lack of awareness. Therefore, the knowledge of this people about maintenance of contact lenses is very critical. The presence of other microorganisms accompanied with *Acanthamoeba* in contact lenses has a considerable role in the increase of number of trophozoite adhesion to lenses and thus the increase prevalence of *Acanthamoeba* keratitis (10). Health care facilities and hospitals supply their water from city water system and/or from their own storage tanks. These could be potential sources for nosocomial infections caused by *Acanthamoeba* (11). Given the fact that in-ward patients need especial care to prevent nosocomial infections, in addition to some reports on *Acanthamoeba* infections (12–17), investigation of drinking water of hospitals has become the focus of attention.

A few studies conducted in Iran have focused on tracing *Acanthamoeba* in environmental specimens (18–20). Moreover, considering there was no similar study in Iran, this study has been conducted to investigate the presence of *Acanthamoeba* in potable water from tap-water at hospitals in several cities. Determining of *Acanthamoeba* carried out in the base of diagnostic characteristics of trophozoite and cyst, particularly the shape of double-layered cell wall of cyst.

## Materials and Methods

This descriptive cross-sectional study took 7 months to be completed, from December 2007 to June 2008. Ninety-four samples were collected from warm and cold tap-water of hospitals in 14 cities including Mashhad, Tehran, Esfahan, Tabriz, Shiraz, Ahvaz, Arak, Hamedan, Sari, Rasht, Shahre kord, Boshehr, Semnan and Orumiyeh. All samples were put into 4-litre plastic containers, and were carried to a Parasitology lab in Baqiyatallah University of Medical Sciences, Tehran, Iran. At the time of sampling, pH, free residual chlorine, and temperature were measured with pH and Cl D.P.D Amcor test Kit. After filtrations by multi pore nylon cellulose nitrate membrane (Filter pore size, 0.22– 0.45µm), the filter was separated from filtration and then was placed on the non-nutrient agar medium directly. Non-nutrient agar (NNA) was prepared with Amoeba Page Saline (20). To enrich the cultural media and provide *Acanthamoeba* food, we added some heat-killed *Escherichia coli* with special consideration. That *E. coli* was in the form of suspension, which had been placed in 56°C Ban-mary for 20 minutes (21).

0.1ml of water samples, which was prepared before, was placed on sterile condition in cultural media. It was kept in the room temperature. After two weeks, the plates were monitored daily for the outgrowth of *Acanthamoeba*. This was repeated for 10 days. To investigate the presence of *Acanthamoeba*, we used reversed contrast phase microscope with a 10x objective. Protozoa grow up in cultural media. We found them for their trophozoite and cystic characteristics, especially two-layer cell wall cysts.

## Results

**Fig. 1:**
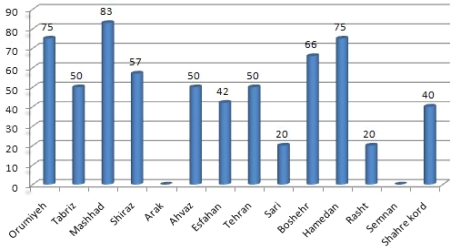
Percent of *Acanthamoeba* isolated from samples of drinking water in some hospitals in Iran

Based on morphological characteristics of trophozoite and cysts, in total, 45 samples (48%) appeared to have developed *Acanthamoeba*. Fig. 1 shows the percentage of positive samples for each city. There was no positive sample in Arak and Semnan hospitals. Table 1 shows the physical characterizations of water collected and cases of *Acanthamoeba* cultivated. The range of temperature of samples was 20–48°C. The highest and the lowest temperature for positive samples were 48°C and 21°C, respectively. The highest and the lowest pH value for positive cases were 7.1 (in Tehran) and 6 (in Rasht), respectively.

**Table 1:** Physical characterizations of water collected and cases of *Acanthamoeba* cultivated

	Type of sample		pH		Free residual chlorine (ppm)			Temperature (°C)		
	
	Warm	Cold	6–7	7.1–8	0.2>	0.2	0.2<	20–30	31–40	41–50
**No. of samples**	21	73	89	5	13	61	20	78	8	8
**Positive cases**	11	34	43	2	7	30	8	35	5	5
**Percentage**	52.4	46.6	48.3	40	53.8	49.2	40	44.9	62.5	62.5

*Acanthamoeba* isolated from samples of drinking water in some hospitals in Iran.

## Discussion

Survival and extension of *Acanthamoeba* in the nature and especially in water demonstrates the potential capability of pathogenesis of this protozoan in both human and animal (1). Many studies have reported the presence of *Acanthamoeba* in drinking water, swimming pools, and rivers. These water sources have an obvious role in prevalence *Acanthamoeba* keratitis among people. Since *Acanthamoeba* has an extensive dispersion, it is expected that individuals have exposure to the protozoa (19, 20, 22–26). For this reason, it has been claimed that more than 80% of healthy people have antibodies against *Acanthamoeba* (27).

Previous studies have shown that many *Acanthamoeba* isolated from tap-water sources might have some pathogenic ability (24, 25). Water distribution systems as well as water tanks in cities have a major role on spreading this protozoon. To meet emergency requirements, hospitals generally have water tanks (11). Patients are also among susceptible people who are more likely to take infection of *Acanthamoeba* in hospitals. Water piping in hospitals is often old and partially somewhat damaged. Metal traces, organic pollutant in drinking water, water with high hardness and free chlorine, low or high pH could lead to adverse health effects. Domestic tap water, especially when supplied from roof storage tanks, is a source of *Acanthamoeba* contamination. People who use contact lens should be aware of the risks associated with *Acanthamoeba* in tap water supplied from water storage tanks (23).

In this study, that was conducted for the first time in Iran, the results showed that about half samples were infected with *Acanthamoeba*. This result demonstrates the considerable spreading of *Acanthamoeba* in different regions among the country. This can be due to high resistance of *Acanthamoeba* against water chlorination as well as tolerance of it to the relatively high temperature. In a study, the minimum concentration of 1.5 parts per million (ppm), free residual chlorine found to be effective against *Acanthamoeba* cyst (26). In another study, it has been reported that some strains of this protozoon were able to survive and grew up, however could not cause illness (22). In Spain, *Acanthamoeba* contamination was found in 48 out of 88 (59.5%) tap water samples (25). The results of this study are consistent with the similar studies conducted elsewhere (19, 23, 25, 28).

*Acanthamoeba* in drinking water supply systems could contaminate contact lenses and eventually lead to *Acanthamoeba* keratitis. In addition, entering human body via respiratory system can cause infection in healthy individuals as well as in persons with immunodeficiency in hospitals. Taking into consideration the reported health-care infections in different wards of hospitals (12–16), some isolated cases extracted from public water distribution systems (25, 29) and storing large amount of water in tanks for a long time in hospitals may possibly be a potential source of spreading of *Acanthamoeba* infection. In this sort of hospitals, patients due to their exceptional conditions are more susceptible to the infection.

Systemic infections of *Acanthamoeba* are usually found among patients with immunodeficiency and receptors of suppressor's drug for chemical therapy. However, normal persons may also be infected. Considering the increase of patients with immunodeficiency system and particularly the patients admitted in kidney transplant, Dialysis, CCU, intensive care unit (ICU), children, and infection wards, the increase of *Acanthamoeba* infection of CNS can be expected. Amoeba-associated bacteria as agents of ventilator-associated pneumonia (VAP), in ICU, especially when microbiologic investigations are negative (14). Specimens from 12 (40%) of 30 patients in an ICU seroconverted to microorganisms known to survive in an aquatic environment in the intracellular niche provided by free-living *Acanthamoeba* (14). These seroconversions were associated with ventilator-associated pneumonia, especially in patients who showed no etiologic agent by usual microbiologic investigations. These results show the role of different *Acanthamoeba* species as a principal co-factor and/or as main source of infections (1). Nevertheless, increase of number of reported cases is mainly due to increase of diagnoses; however, this type of infection is multifactor.

The comparison of finding of this study with similar studies conducted in overseas, shows that *Acanthamoeba* contamination rate is relatively lower in Iran. This could be justified by small number of samples and/or broad location of study.

These results demonstrate that domestic tap water is a significant source of the organisms. In conclusion, to understand the precise spreading of this protozoon in the environment and related factors for its pathogenesis as well as planning for control and prevention, further studies are required. More education about the hygienic maintenance of water storage tanks is recommended.

## References

[1] Marciano-Cabral F, Cabral G (2003). *Acanthamoeba* spp. as agents of disease in humans. Clin Microbiol Rev.

[2] Edrisian GH, Rezaian M, Ghorbani M, Keshavarz H, Mohebali M (2008). Medical protozology.

[3] King CH, Shotts EB, Wooley RE, Porter KG (1988). Survival of coliforms and bacterial pathogens within protozoa during chlorination. Appl Environ Microbiol.

[4] Michel R, Hauroder B (1997). Isolation of an Acanthamoeba strain with intracellular Burkholderia pickettii infection. Zentralbl Bakteriol.

[5] Rowbotham TJ (1980). Preliminary report on the pathogenicity of *Legionella pneumophila* for freshwater and soil amoebae. J Clin Pathol.

[6] Culbertson CG, Smith JW, Cohen HK, Minner JR (1959). Experimental infection of mice and monkeys by *Acanthamoeba*. Am J Pathol.

[7] Steinberg J, Galindo R, Kraus E, Ghanem K (2002). Disseminated acanthamoebiasis in a renal transplant recipient with osteom-yelitis and cutaneous lesions: case report and literature review. Clin Infect Dis.

[8] Parija SC, Prakash MR, Rao VA, Vellaniparambil RJ (2001). *Acanthamoeba* keratitis in Pondicherry. J Commun Dis.

[9] Stehr-Green JK, Bailey TM, Visvesvara GS (1989). The epidemiology of *Acanthamoeba* keratitis in the United States. Am J Ophthalmol.

[10] Winiecka-Krusnell J, Linder E (2001). Bacterial infections of free-living amoebae. Res Microbiol.

[11] Michel R, Burghardt H, Bergmann H (1995). *Acanthamoeba*, naturally intracellularly infected with *Pseudomonas aeruginosa*, after their isolation from a microbi-ologically contaminated drinking water system in a hospital. Zentralbl Hyg Umweltmed.

[12] Anaissie EJ, Penzak SR, Dignani MC (2002). The hospital water supply as a source of nosocomial infections: a plea for action. Arch Intern Med.

[13] Cook D (2000). Ventilator associated pneumonia: perspectives on the burden of illness. Intensive Care Med.

[14] La Scola B, Boyadjiev I, Greub G, Khamis A, Martin C, Raoult D (2003). Amoeba-resisting bacteria and ventilator-associated pneumonia. Emerg Infect Dis.

[15] La Scola B, Mezi L, Auffray JP, Berland Y, Raoult D (2002). Patients in the intensive care unit are exposed to amoeba-associated pathogens. Infect Control Hosp Epidemiol.

[16] Marrie TJ, Raoult D, La Scola B, Birtles RJ, de Carolis E (2001). *Legionella*-like and other amoebal pathogens as agents of community-acquired pneumonia. Emerg Infect Dis.

[17] Berger P, Papazian L, Drancourt M, La Scola B, Auffray JP, Raoult D (2006). Ameba-associated microorganisms and diagnosis of nosocomial pneumonia. Emerg Infect Dis.

[18] Maghsood AH, Sissons J, Rezaian M, Nolder D, Warhurst D, Khan NA (2005). *Acanthamoeba* genotype T4 from the UK and Iran and isolation of the T2 genotype from clinical isolates. J Med Microbiol.

[19] Niyyati M, Lorenzo-Morales J, Rezaie S, Rahimi F, Mohebali M, Maghsood AH (2009). Genotyping of *Acanthamoeba* isolates from clinical and environmental speci-mens in Iran. Exp Parasitol.

[20] Rezaeian M, Niyyati M, Farnia S, Haghi AM (2008). Isolation of *Acanthamoeba spp*. from Different Environmental Sources. Iranian J Parasitol.

[21] Schuster FL (2002). Cultivation of pathogenic and opportunistic free-living amebas. Clin Microbiol Rev.

[22] De Jonckheere JF (1979). Pathogenic free-living amoebae in swimming pools: survey in Belgium. Ann Microbiol.

[23] Jeong HJ, Yu HS (2005). The role of domestic tap water in Acanthamoeba contamination in contact lens storage cases in Korea. Korean J Parasitol.

[24] Kilvington S, Gray T, Dart J, Morlet N, Beeching JR, Frazer DG (2004). *Acanthamoeba* keratitis: the role of domestic tap water contamination in the United Kingdom. Invest Ophthalmol Vis Sci.

[25] Lorenzo-Morales J, Lindo JF, Martinez E, Calder D, Figueruelo E, Valladares B (2005). Pathogenic *Acanthamoeba* strains from water sources in Jamaica, West Indies. Ann Trop Med Parasitol.

[26] Rivera F, Ramirez E, Bonilla P, Calderon A, Gallegos E, Rodriguez S (1993). Pathogenic and free-living amoebae isolated from swimming pools and physiotherapy tubs in Mexico. Environ Res.

[27] Chappell CL, Wright JA, Coletta M, Newsome AL (2001). Standardized method of measuring *Acanthamoeba* antibodies in sera from healthy human subjects. Clin Diagn Lab Immunol.

[28] Al-Herrawy AZ, Al-Rasheid KA (1998). Identi-fication of *Acanthamoeba* strains isolated from a freshwater course in Saudi Arabia. J Egypt Public Health Assoc.

[29] Lorenzo-Morales J, Ortega-Rivas A, Foronda P, Martinez E, Valladares B (2005). Isolation and identification of pathogenic *Acanthamoeba* strains in Tenerife, Canary Islands, Spain from water sources. Parasitol Res.

